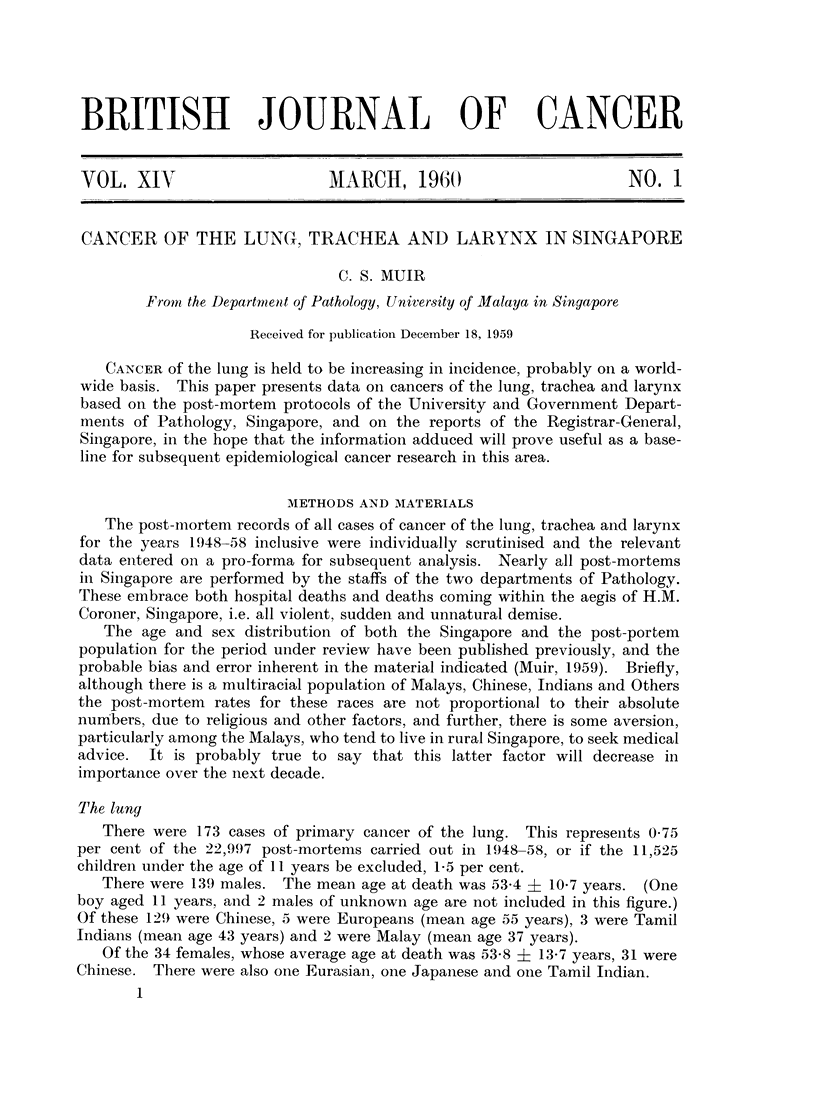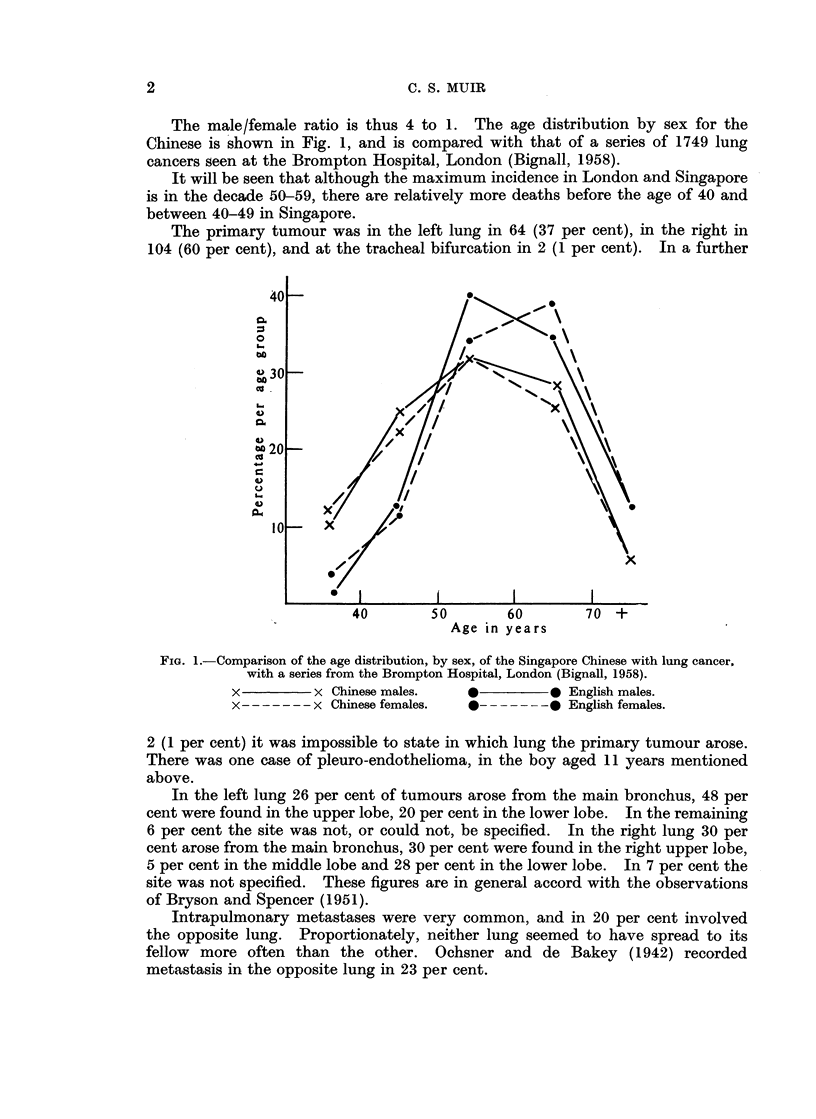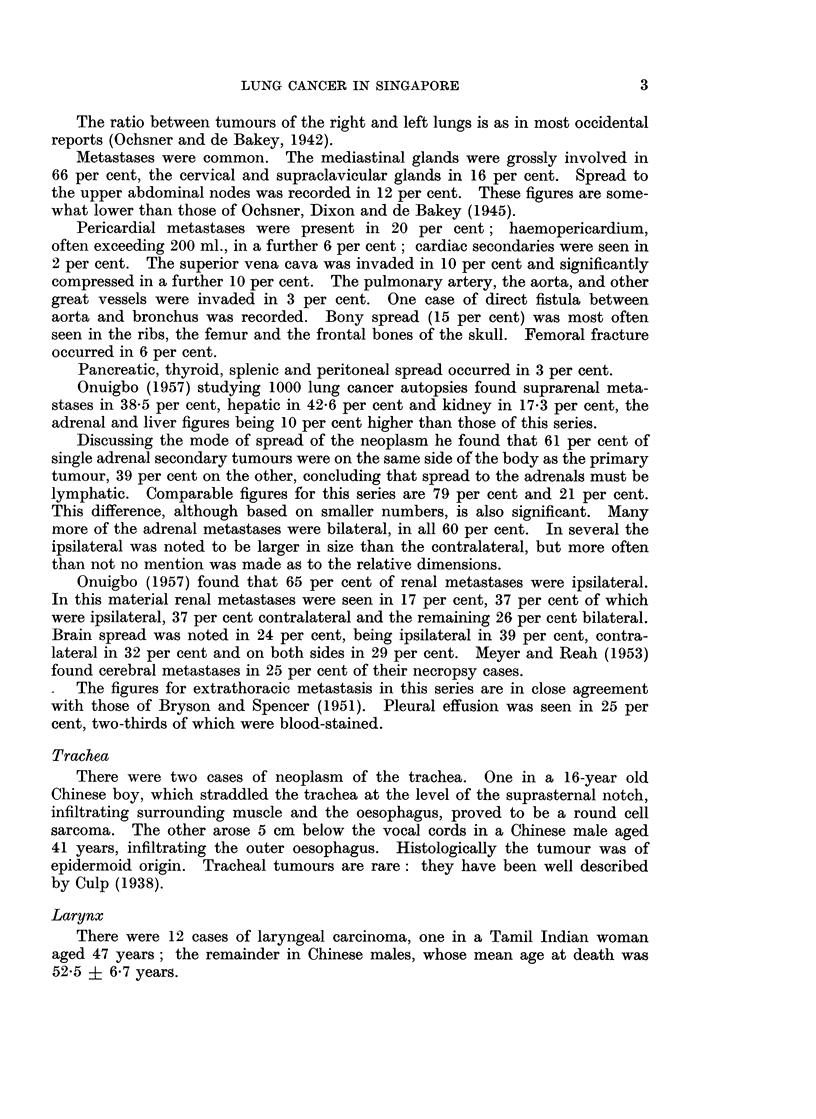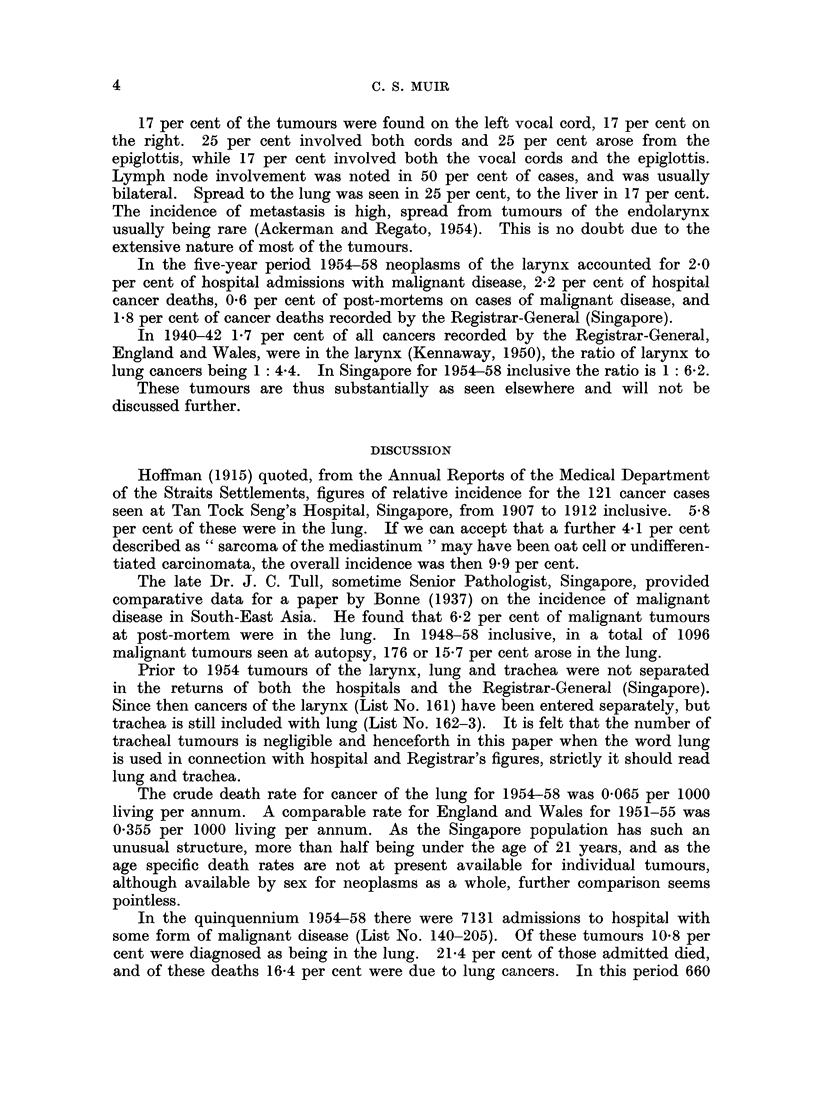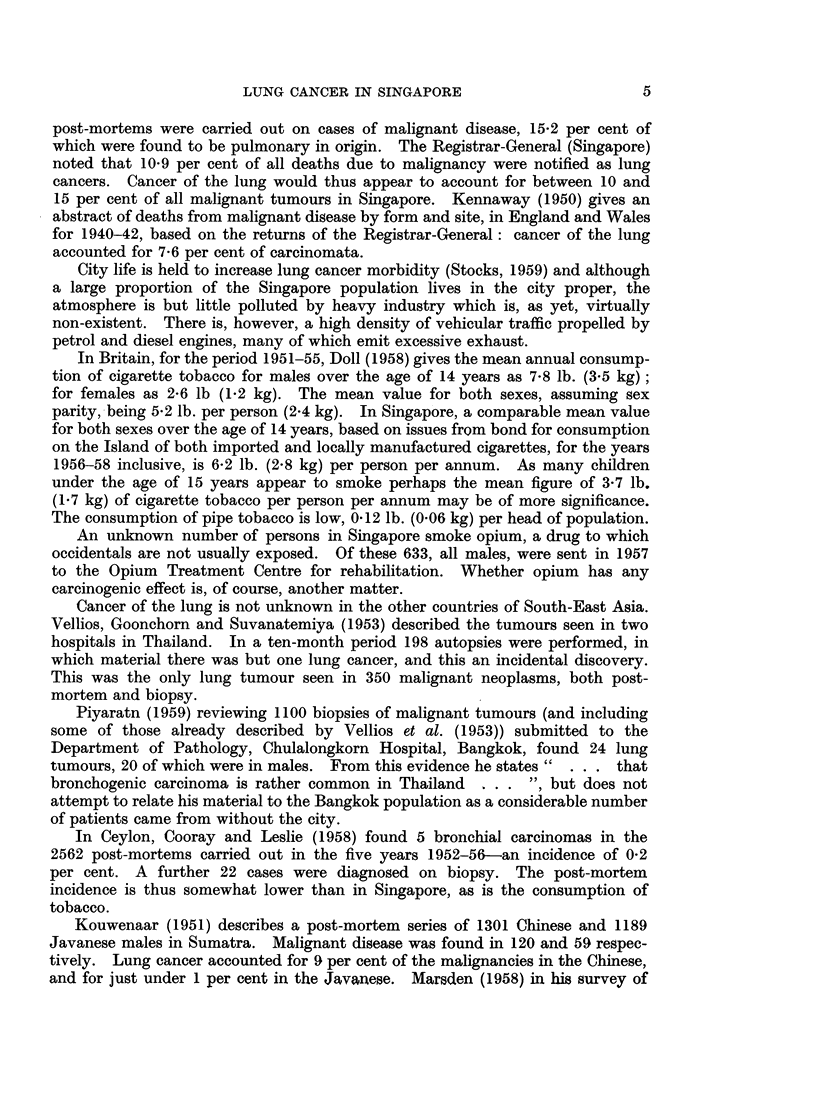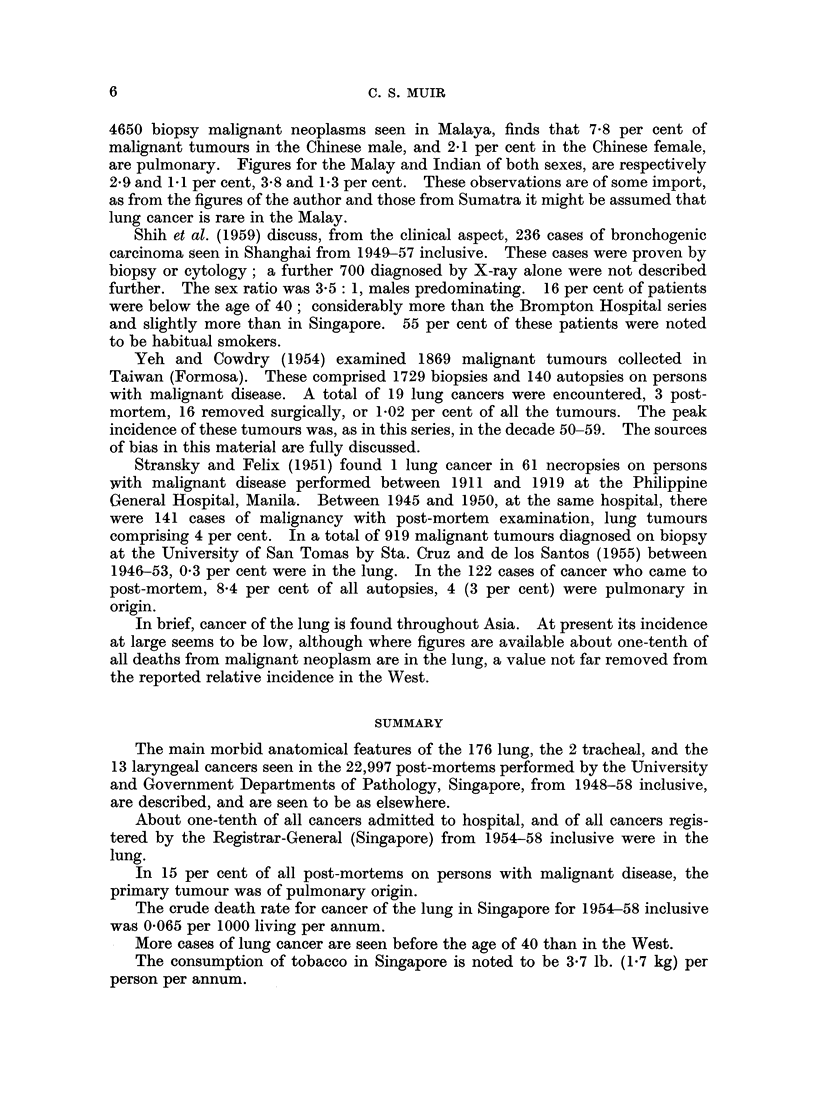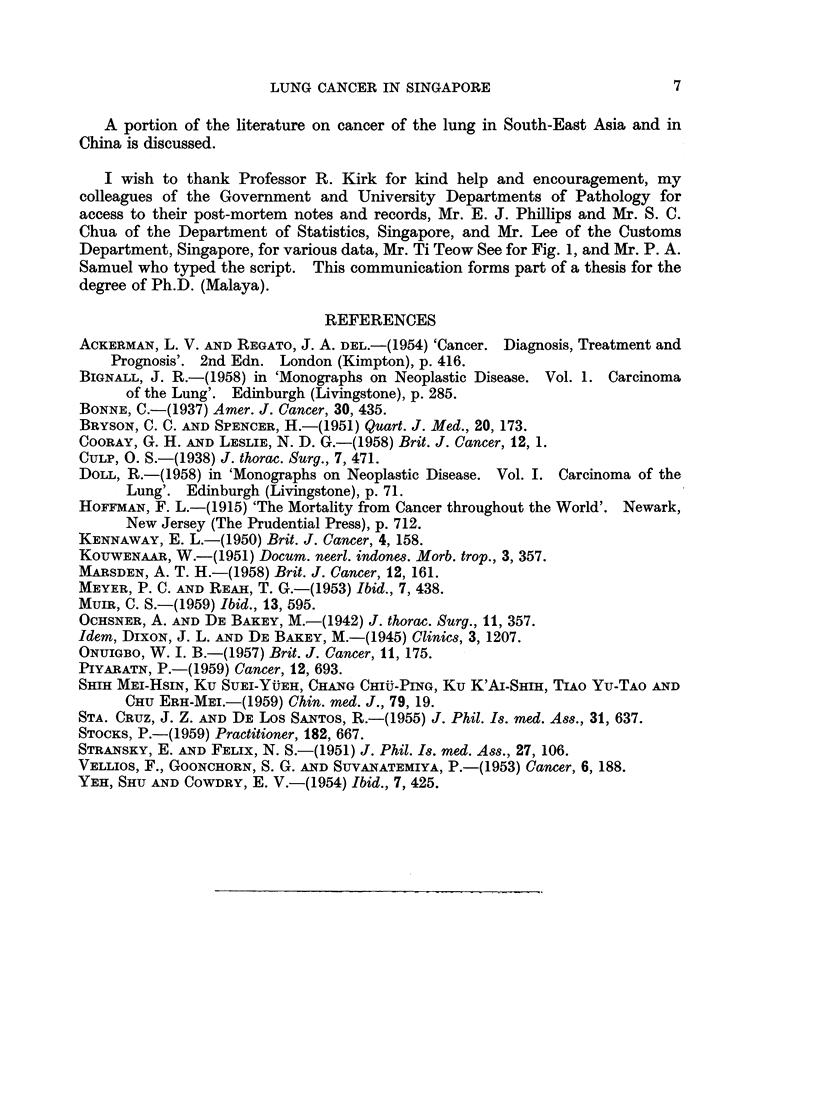# Cancer of the Lung, Trachea and Larynx in Singapore

**DOI:** 10.1038/bjc.1960.1

**Published:** 1960-03

**Authors:** C. S. Muir


// BRITISH JOURNAL OF CANCER

VOL. XIV MARCH, 1960 NO. 1

### CANCER OF THE LUNG, TRACHEA AND LARYNX IN SINGAPORE

C. S. MUIR

*From the Department of Pathology, University of Malaya in Singapore*

Received for publication December 18, 1959

CANCER of the lung is held to be increasing in incidence, probably on a world-wide basis. This paper presents data on cancers of the lung, trachea and larynx based on the post-mortem protocols of the University and Government Departments of Pathology, Singapore, and on the reports of the Registrar-General, Singapore, in the hope that the information adduced will prove useful as a base-line for subsequent epidemiological cancer research in this area.

### METHODS AND MATERIALS

The post-mortem records of all cases of cancer of the lung, trachea and larynx for the years 1948–58 inclusive were individually scrutinised and the relevant data entered on a pro-forma for subsequent analysis. Nearly all post-mortems in Singapore are performed by the staffs of the two departments of Pathology. These embrace both hospital deaths and deaths coming within the aegis of H.M. Coroner, Singapore, i.e. all violent, sudden and unnatural demise.

The age and sex distribution of both the Singapore and the post-portem population for the period under review have been published previously, and the probable bias and error inherent in the material indicated (Muir, 1959). Briefly, although there is a multiracial population of Malays, Chinese, Indians and Others the post-mortem rates for these races are not proportional to their absolute numbers, due to religious and other factors, and further, there is some aversion, particularly among the Malays, who tend to live in rural Singapore, to seek medical advice. It is probably true to say that this latter factor will decrease in importance over the next decade.

### *The lung*

There were 173 cases of primary cancer of the lung. This represents 0·75 per cent of the 22,997 post-mortems carried out in 1948–58, or if the 11,525 children under the age of 11 years be excluded, 1·5 per cent.

There were 139 males. The mean age at death was 53·4 ± 10·7 years. (One boy aged 11 years, and 2 males of unknown age are not included in this figure.) Of these 129 were Chinese, 5 were Europeans (mean age 55 years), 3 were Tamil Indians (mean age 43 years) and 2 were Malay (mean age 37 years).

Of the 34 females, whose average age at death was 53·8 ± 13·7 years, 31 were Chinese. There were also one Eurasian, one Japanese and one Tamil Indian.

The male/female ratio is thus 4 to 1. The age distribution by sex for the Chinese is shown in Fig. 1, and is compared with that of a series of 1749 lung cancers seen at the Brompton Hospital, London (Bignall, 1958).

It will be seen that although the maximum incidence in London and Singapore is in the decade 50–59, there are relatively more deaths before the age of 40 and between 40–49 in Singapore.

The primary tumour was in the left lung in 64 (37 per cent), in the right in 104 (60 per cent), and at the tracheal bifurcation in 2 (1 per cent). In a further

FIG. 1.—Comparison of the age distribution, by sex, of the Singapore Chinese with lung cancer, with a series from the Brompton Hospital, London (Bignall, 1958).

×————× Chinese males. ●————● English males.

×– – – – ×Chinese females. ●– – – –● English females.

2 (1 per cent) it was impossible to state in which lung the primary tumour arose. There was one case of pleuro-endothelioma, in the boy aged 11 years mentioned above.

In the left lung 26 per cent of tumours arose from the main bronchus, 48 per cent were found in the upper lobe, 20 per cent in the lower lobe. In the remaining 6 per cent the site was not, or could not, be specified. In the right lung 30 per cent arose from the main bronchus, 30 per cent were found in the right upper lobe, 5 per cent in the middle lobe and 28 per cent in the lower lobe. In 7 per cent the site was not specified. These figures are in general accord with the observations of Bryson and Spencer (1951).

Intrapulmonary metastases were very common, and in 20 per cent involved the opposite lung. Proportionately, neither lung seemed to have spread to its fellow more often than the other. Ochsner and de Bakey (1942) recorded metastasis in the opposite lung in 23 per cent.

The ratio between tumours of the right and left lungs is as in most occidental reports (Ochsner and de Bakey, 1942).

Metastases were common. The mediastinal glands were grossly involved in 66 per cent, the cervical and supraclavicular glands in 16 per cent. Spread to the upper abdominal nodes was recorded in 12 per cent. These figures are somewhat lower than those of Ochsner, Dixon and de Bakey (1945).

Pericardial metastases were present in 20 per cent ; haemopericardium, often exceeding 200 ml., in a further 6 per cent ; cardiac secondaries were seen in 2 per cent. The superior vena cava was invaded in 10 per cent and significantly compressed in a further 10 per cent. The pulmonary artery, the aorta, and other great vessels were invaded in 3 per cent. One case of direct fistula between aorta and bronchus was recorded. Bony spread (15 per cent) was most often seen in the ribs, the femur and the frontal bones of the skull. Femoral fracture occurred in 6 per cent.

Pancreatic, thyroid, splenic and peritoneal spread occurred in 3 per cent.

Onuigbo (1957) studying 1000 lung cancer autopsies found suprarenal metastases in 38·5 per cent, hepatic in 42·6 per cent and kidney in 17·3 per cent, the adrenal and liver figures being 10 per cent higher than those of this series.

Discussing the mode of spread of the neoplasm he found that 61 per cent of single adrenal secondary tumours were on the same side of the body as the primary tumour, 39 per cent on the other, concluding that spread to the adrenals must be lymphatic. Comparable figures for this series are 79 per cent and 21 per cent. This difference, although based on smaller numbers, is also significant. Many more of the adrenal metastases were bilateral, in all 60 per cent. In several the ipsilateral was noted to be larger in size than the contralateral, but more often than not no mention was made as to the relative dimensions.

Onuigbo (1957) found that 65 per cent of renal metastases were ipsilateral. In this material renal metastases were seen in 17 per cent, 37 per cent of which were ipsilateral, 37 per cent contralateral and the remaining 26 per cent bilateral. Brain spread was noted in 24 per cent, being ipsilateral in 39 per cent, contralateral in 32 per cent and on both sides in 29 per cent. Meyer and Reah (1953) found cerebral metastases in 25 per cent of their necropsy cases.

The figures for extrathoracic metastasis in this series are in close agreement with those of Bryson and Spencer (1951). Pleural effusion was seen in 25 per cent, two-thirds of which were blood-stained.

*Trachea*

There were two cases of neoplasm of the trachea. One in a 16-year old Chinese boy, which straddled the trachea at the level of the suprasternal notch, infiltrating surrounding muscle and the oesophagus, proved to be a round cell sarcoma. The other arose 5 cm below the vocal cords in a Chinese male aged 41 years, infiltrating the outer oesophagus. Histologically the tumour was of epidermoid origin. Tracheal tumours are rare : they have been well described by Culp (1938).

*Larynx*

There were 12 cases of laryngeal carcinoma, one in a Tamil Indian woman aged 47 years ; the remainder in Chinese males, whose mean age at death was 52·5 $\pm$ 6·7 years.

17 per cent of the tumours were found on the left vocal cord, 17 per cent on the right. 25 per cent involved both cords and 25 per cent arose from the epiglottis, while 17 per cent involved both the vocal cords and the epiglottis. Lymph node involvement was noted in 50 per cent of cases, and was usually bilateral. Spread to the lung was seen in 25 per cent, to the liver in 17 per cent. The incidence of metastasis is high, spread from tumours of the endolarynx usually being rare (Ackerman and Regato, 1954). This is no doubt due to the extensive nature of most of the tumours.

In the five-year period 1954–58 neoplasms of the larynx accounted for 2·0 per cent of hospital admissions with malignant disease, 2·2 per cent of hospital cancer deaths, 0·6 per cent of post-mortems on cases of malignant disease, and 1·8 per cent of cancer deaths recorded by the Registrar-General (Singapore).

In 1940–42 1·7 per cent of all cancers recorded by the Registrar-General, England and Wales, were in the larynx (Kennaway, 1950), the ratio of larynx to lung cancers being 1 : 4·4. In Singapore for 1954–58 inclusive the ratio is 1 : 6·2.

These tumours are thus substantially as seen elsewhere and will not be discussed further.

### DISCUSSION

Hoffman (1915) quoted, from the Annual Reports of the Medical Department of the Straits Settlements, figures of relative incidence for the 121 cancer cases seen at Tan Tock Seng's Hospital, Singapore, from 1907 to 1912 inclusive. 5·8 per cent of these were in the lung. If we can accept that a further 4·1 per cent described as " sarcoma of the mediastinum " may have been oat cell or undifferentiated carcinomata, the overall incidence was then 9·9 per cent.

The late Dr. J. C. Tull, sometime Senior Pathologist, Singapore, provided comparative data for a paper by Bonne (1937) on the incidence of malignant disease in South-East Asia. He found that 6·2 per cent of malignant tumours at post-mortem were in the lung. In 1948–58 inclusive, in a total of 1096 malignant tumours seen at autopsy, 176 or 15·7 per cent arose in the lung.

Prior to 1954 tumours of the larynx, lung and trachea were not separated in the returns of both the hospitals and the Registrar-General (Singapore). Since then cancers of the larynx (List No. 161) have been entered separately, but trachea is still included with lung (List No. 162–3). It is felt that the number of tracheal tumours is negligible and henceforth in this paper when the word lung is used in connection with hospital and Registrar's figures, strictly it should read lung and trachea.

The crude death rate for cancer of the lung for 1954–58 was 0·065 per 1000 living per annum. A comparable rate for England and Wales for 1951–55 was 0·355 per 1000 living per annum. As the Singapore population has such an unusual structure, more than half being under the age of 21 years, and as the age specific death rates are not at present available for individual tumours, although available by sex for neoplasms as a whole, further comparison seems pointless.

In the quinquennium 1954–58 there were 7131 admissions to hospital with some form of malignant disease (List No. 140–205). Of these tumours 10·8 per cent were diagnosed as being in the lung. 21·4 per cent of those admitted died, and of these deaths 16·4 per cent were due to lung cancers. In this period 660

post-mortems were carried out on cases of malignant disease, 15·2 per cent of which were found to be pulmonary in origin. The Registrar-General (Singapore) noted that 10·9 per cent of all deaths due to malignancy were notified as lung cancers. Cancer of the lung would thus appear to account for between 10 and 15 per cent of all malignant tumours in Singapore. Kennaway (1950) gives an abstract of deaths from malignant disease by form and site, in England and Wales for 1940–42, based on the returns of the Registrar-General : cancer of the lung accounted for 7·6 per cent of carcinomata.

City life is held to increase lung cancer morbidity (Stocks, 1959) and although a large proportion of the Singapore population lives in the city proper, the atmosphere is but little polluted by heavy industry which is, as yet, virtually non-existent. There is, however, a high density of vehicular traffic propelled by petrol and diesel engines, many of which emit excessive exhaust.

In Britain, for the period 1951–55, Doll (1958) gives the mean annual consumption of cigarette tobacco for males over the age of 14 years as 7·8 lb. (3·5 kg) ; for females as 2·6 lb (1·2 kg). The mean value for both sexes, assuming sex parity, being 5·2 lb. per person (2·4 kg). In Singapore, a comparable mean value for both sexes over the age of 14 years, based on issues from bond for consumption on the Island of both imported and locally manufactured cigarettes, for the years 1956–58 inclusive, is 6·2 lb. (2·8 kg) per person per annum. As many children under the age of 15 years appear to smoke perhaps the mean figure of 3·7 lb. (1·7 kg) of cigarette tobacco per person per annum may be of more significance. The consumption of pipe tobacco is low, 0·12 lb. (0·06 kg) per head of population.

An unknown number of persons in Singapore smoke opium, a drug to which occidentals are not usually exposed. Of these 633, all males, were sent in 1957 to the Opium Treatment Centre for rehabilitation. Whether opium has any carcinogenic effect is, of course, another matter.

Cancer of the lung is not unknown in the other countries of South-East Asia. Vellios, Goonchorn and Suvanatemiya (1953) described the tumours seen in two hospitals in Thailand. In a ten-month period 198 autopsies were performed, in which material there was but one lung cancer, and this an incidental discovery. This was the only lung tumour seen in 350 malignant neoplasms, both post-mortem and biopsy.

Piyaratn (1959) reviewing 1100 biopsies of malignant tumours (and including some of those already described by Vellios *et al.* (1953)) submitted to the Department of Pathology, Chulalongkorn Hospital, Bangkok, found 24 lung tumours, 20 of which were in males. From this evidence he states " . . . that bronchogenic carcinoma is rather common in Thailand . . . ", but does not attempt to relate his material to the Bangkok population as a considerable number of patients came from without the city.

In Ceylon, Cooray and Leslie (1958) found 5 bronchial carcinomas in the 2562 post-mortems carried out in the five years 1952–56—an incidence of 0·2 per cent. A further 22 cases were diagnosed on biopsy. The post-mortem incidence is thus somewhat lower than in Singapore, as is the consumption of tobacco.

Kouwenaar (1951) describes a post-mortem series of 1301 Chinese and 1189 Javanese males in Sumatra. Malignant disease was found in 120 and 59 respectively. Lung cancer accounted for 9 per cent of the malignancies in the Chinese, and for just under 1 per cent in the Javanese. Marsden (1958) in his survey of

4650 biopsy malignant neoplasms seen in Malaya, finds that 7·8 per cent of malignant tumours in the Chinese male, and 2·1 per cent in the Chinese female, are pulmonary. Figures for the Malay and Indian of both sexes, are respectively 2·9 and 1·1 per cent, 3·8 and 1·3 per cent. These observations are of some import, as from the figures of the author and those from Sumatra it might be assumed that lung cancer is rare in the Malay.

Shih *et al.* (1959) discuss, from the clinical aspect, 236 cases of bronchogenic carcinoma seen in Shanghai from 1949–57 inclusive. These cases were proven by biopsy or cytology ; a further 700 diagnosed by X-ray alone were not described further. The sex ratio was 3·5 : 1, males predominating. 16 per cent of patients were below the age of 40 ; considerably more than the Brompton Hospital series and slightly more than in Singapore. 55 per cent of these patients were noted to be habitual smokers.

Yeh and Cowdry (1954) examined 1869 malignant tumours collected in Taiwan (Formosa). These comprised 1729 biopsies and 140 autopsies on persons with malignant disease. A total of 19 lung cancers were encountered, 3 post-mortem, 16 removed surgically, or 1·02 per cent of all the tumours. The peak incidence of these tumours was, as in this series, in the decade 50–59. The sources of bias in this material are fully discussed.

Stransky and Felix (1951) found 1 lung cancer in 61 necropsies on persons with malignant disease performed between 1911 and 1919 at the Philippine General Hospital, Manila. Between 1945 and 1950, at the same hospital, there were 141 cases of malignancy with post-mortem examination, lung tumours comprising 4 per cent. In a total of 919 malignant tumours diagnosed on biopsy at the University of San Tomas by Sta. Cruz and de los Santos (1955) between 1946–53, 0·3 per cent were in the lung. In the 122 cases of cancer who came to post-mortem, 8·4 per cent of all autopsies, 4 (3 per cent) were pulmonary in origin.

In brief, cancer of the lung is found throughout Asia. At present its incidence at large seems to be low, although where figures are available about one-tenth of all deaths from malignant neoplasm are in the lung, a value not far removed from the reported relative incidence in the West.

### SUMMARY

The main morbid anatomical features of the 176 lung, the 2 tracheal, and the 13 laryngeal cancers seen in the 22,997 post-mortems performed by the University and Government Departments of Pathology, Singapore, from 1948–58 inclusive, are described, and are seen to be as elsewhere.

About one-tenth of all cancers admitted to hospital, and of all cancers registered by the Registrar-General (Singapore) from 1954–58 inclusive were in the lung.

In 15 per cent of all post-mortems on persons with malignant disease, the primary tumour was of pulmonary origin.

The crude death rate for cancer of the lung in Singapore for 1954–58 inclusive was 0·065 per 1000 living per annum.

More cases of lung cancer are seen before the age of 40 than in the West.

The consumption of tobacco in Singapore is noted to be 3·7 lb. (1·7 kg) per person per annum.

A portion of the literature on cancer of the lung in South-East Asia and in China is discussed.

I wish to thank Professor R. Kirk for kind help and encouragement, my colleagues of the Government and University Departments of Pathology for access to their post-mortem notes and records, Mr. E. J. Phillips and Mr. S. C. Chua of the Department of Statistics, Singapore, and Mr. Lee of the Customs Department, Singapore, for various data, Mr. Ti Teow See for Fig. 1, and Mr. P. A. Samuel who typed the script. This communication forms part of a thesis for the degree of Ph.D. (Malaya).

### REFERENCES

ACKERMAN, L. V. AND REGATO, J. A. DEL.—(1954) 'Cancer. Diagnosis, Treatment and Prognosis'. 2nd Edn. London (Kimpton), p. 416.
BIGNALL, J. R.—(1958) in 'Monographs on Neoplastic Disease. Vol. 1. Carcinoma of the Lung'. Edinburgh (Livingstone), p. 285.
BONNE, C.—(1937) *Amer. J. Cancer*, **30**, 435.
BRYSON, C. C. AND SPENCER, H.—(1951) *Quart. J. Med.*, **20**, 173.
COORAY, G. H. AND LESLIE, N. D. G.—(1958) *Brit. J. Cancer*, **12**, 1.
CULP, O. S.—(1938) *J. thorac. Surg.*, **7**, 471.
DOLL, R.—(1958) in 'Monographs on Neoplastic Disease. Vol. I. Carcinoma of the Lung'. Edinburgh (Livingstone), p. 71.
HOFFMAN, F. L.—(1915) 'The Mortality from Cancer throughout the World'. Newark, New Jersey (The Prudential Press), p. 712.
KENNAWAY, E. L.—(1950) *Brit. J. Cancer*, **4**, 158.
KOUWENAAR, W.—(1951) *Docum. neerl. indones. Morb. trop.*, **3**, 357.
MARSDEN, A. T. H.—(1958) *Brit. J. Cancer*, **12**, 161.
MEYER, P. C. AND REAH, T. G.—(1953) *Ibid.*, **7**, 438.
MUIR, C. S.—(1959) *Ibid.*, **13**, 595.
OCHSNER, A. AND DE BAKEY, M.—(1942) *J. thorac. Surg.*, **11**, 357.
*Idem*, DIXON, J. L. AND DE BAKEY, M.—(1945) *Clinics*, **3**, 1207.
ONUIGBO, W. I. B.—(1957) *Brit. J. Cancer*, **11**, 175.
PIYARATN, P.—(1959) *Cancer*, **12**, 693.
SHIH MEI-HSIN, KU SUEI-YÜEH, CHANG CHIÜ-PING, KU K'AI-SHIH, TIAO YU-TAO AND CHU ERH-MEI.—(1959) *Chin. med. J.*, **79**, 19.
STA. CRUZ, J. Z. AND DE LOS SANTOS, R.—(1955) *J. Phil. Is. med. Ass.*, **31**, 637.
STOCKS, P.—(1959) *Practitioner*, **182**, 667.
STRANSKY, E. AND FELIX, N. S.—(1951) *J. Phil. Is. med. Ass.*, **27**, 106.
VELLIOS, F., GOONCHORN, S. G. AND SUVANATEMIYA, P.—(1953) *Cancer*, **6**, 188.
YEH, SHU AND COWDRY, E. V.—(1954) *Ibid.*, **7**, 425.